# A gated deep inspiration breath‐hold radiation therapy technique using a linear position transducer

**DOI:** 10.1120/jacmp.v6i1.1995

**Published:** 2005-03-17

**Authors:** Svetlana I. Denissova, Mammo H. Yewondwossen, John W. Andrew, Michael E. Hale, Carl H. Murphy, Scott R. Purcell

**Affiliations:** ^1^ QEII Health Sciences Centre Halifax Nova Scotia; ^2^ Department of Radiation Oncology Dalhousie University Halifax Nova Scotia; ^3^ PEI Cancer Treatment Centre Charlottetown Prince Edward Island Canada

**Keywords:** lung cancer, respiratory organ movement, respiratory gating, gated therapy

## Abstract

For patients with thoracic and abdominal lesions, respiration‐induced internal organ motion and deformations during radiation therapy are limiting factors for the administration of high radiation dose. To increase the dose to the tumor and to reduce margins, tumor movement during treatment must be minimized. Currently, several types of breath‐synchronized systems are in use. These systems include respiratory gating, deep inspiration breath‐hold, active breathing control, and voluntary breath‐hold. We used a linear position transducer (LPT) to monitor changes in a patient's abdominal cross‐sectional area. The LPT tracks changes in body circumference during the respiratory cycle using a strap connected to the LPT and wrapped around the patient's torso. The LPT signal is monitored by a computer that provides a real‐time plot of the patient's breathing pattern. In our technique, we use a CT study with multiple gated acquisitions. The Philips Medical Systems Q series CT imaging system is capable of operating in conjunction with a contrast injector. This allows a patient performing the deep inspiration breath‐hold maneuver to send a signal to trigger the CT scanner acquisitions. The LPT system, when interfaced to a LINAC, allows treatment to be delivered only during deep inspiration breath‐hold periods. Treatment stops automatically if the lung volume drops from a preset value. The whole treatment can be accomplished with 1 to 3 breath‐holds. This technique has been used successfully to combine automatically gated radiation delivery with the deep inspiration breath‐hold technique. This improves the accuracy of treatment for moving tumors, providing better target coverage, sparing more healthy tissue, and saving machine time.

PACS numbers: 87.53.2j, 87.57.‐s

## I. INTRODUCTION

Lung cancer is the second most common cancer and considered to be the leading cause of cancer death among both men and women.^(1)^ Surgery is the primary, potentially curative, therapeutic option for lung cancer. However, because of stage and medical condition at presentation, it is estimated that only 20% of patients initially presenting with lung cancer are eligible for definitive surgery.^(2)^ For the remaining 80%, radiation therapy can produce a cure in a small minority and palliation in the majority of patients. The factor most limiting the spatial accuracy of radiation treatment of lung cancer patients is tumor movement during the respiration cycle. In conventional external beam radiotherapy, to cover the tumor motion during breathing, the planning target volume (PTV) includes a margin to account for the respiratory movement.^(3)^ The magnitude of the respiratory movement in the thorax and abdomen is estimated to be up to 3 cm during normal breathing.^(^ ^4^ ^–^ ^7^ ^)^ In cases where there is a considerable range of tumor movement, an attempt to deliver an adequate dose to the tumor will increase the risk of severe normal tissue complications. The ability to localize the tumor precisely during treatment delivery will lead to some improvements in the therapeutic ratio.

Breathing‐synchronized radiotherapy allows the correlation of treatment delivery with the respiratory cycle. As a result, the radiation field can be reduced substantially to avoid normal tissue complications. This allows higher doses to the tumor and improves chances of local tumor control. Currently, several types of breathing‐synchronized systems, based on different types of organ motion detectors, are in use in radiotherapy.^(^ ^4^ ^,^ ^5^ ^,^ ^8^ ^–^ ^12^ ^)^ Tumor motion is tracked using direct (e.g., diagnostic X‐ray imaging) or indirect tumor position detection. The indirect approaches estimate tumor displacement from chest wall movement, monitor air volume in the lungs, and measure airflow temperature variations or changes in a cross‐sectional area of the thorax. Good correlation between the motion of the tumor and the motion of the diaphragm, as monitored by external detectors, has been demonstrated by several authors.^(6)^ ^,^ ^(8)^ ^,^ ^(13)^

The beam can be gated during free breathing and dose delivered at a certain level of respiration, or treatment can be delivered during breath‐hold when the tumor is immobilized and its location can be determined more accurately. Breath‐hold can be controlled voluntarily or using an active breathing control device. The breath‐hold technique is usually performed either at the exhalation phase or the inhalation phase of the respiration cycle. It is believed that the exhalation stage is more reproducible because the diaphragm is more relaxed at this time.^(5)^ ^,^ ^(10)^ ^,^ ^(14)^ In the case of treatment delivery during the inhalation stage, the expansion of the lungs allows for a significant volume of healthy tissue to be driven out of high‐dose areas.

We have developed a new technique for the treatment of lung cancer patients who exhibit a significant range of tumor movement during the respiration cycle. This approach combines the principle of an automatically gated treatment with a deep inspiration breath‐hold (DIBH) maneuver. The deep‐breath‐hold technique reduces the residual tumor motion to approximately 10% compared to free breathing and gating radiation delivery.^(15)^ The suppressed target motion excludes normal lung tissues from the high‐dose region.

## II. MATERIALS AND METHODS

### A. Linear position transducer‐based breath‐hold technique

**Figure 1:**
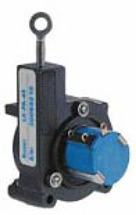
Linear position transducer UniMeasure LX‐PA‐2

Our system is based on the use of a linear position transducer (LPT) LX‐PA‐2 (UniMeasure Inc., U.S.A.) to monitor changes in the patient's abdominal cross‐sectional area caused by breathing. The LPT is essentially a spring‐loaded potentiometer with a light wire rope wound around the axis of the potentiometer (Fig. 1).

**Figure 2:**
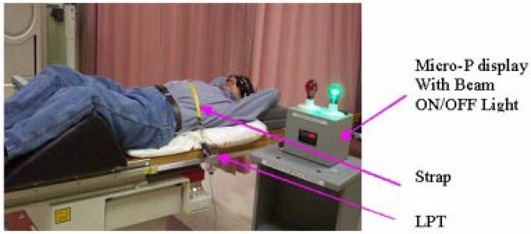
Patient setup showing the strap around the patient's chest and the LPT attached to the railing of the bed

The LPT is fixed to one railing of the treatment bed next to the patient's abdomen (Fig. 2). A light strap, fixed at one end to the other bed rail, runs over the patient's upper abdominal region and is connected to the wire rope from the LPT. The LPT is used to generate a voltage signal from the changing circumference in the upper abdominal region as the patient breathes. This device detects a linear range of motion in the strap of 50 mm at a nominal output of 18.5mV/mm and a nominal wire rope tension of 4.4 N. Weight loss does not affect the gating signal from the LPT because the LPT voltage window is adjusted for a deep inspiration maneuver and accounts only for the difference between the deep inspiration and normal breathing level.

The LPT analog signals are sent to a Micro‐P display, which acts as the processing interface between the analog LPT and the user. It applies a current to the resistor in the LPT and displays the measured voltage on the LED display. Every millisecond, it reads the value from the LPT and automatically displays the reading value on the LED display. An RS‐232 serial port allows the Micro‐P to communicate with a computer, enabling the computer to graph the chest movement in real time at the treatment console so that the therapist can observe the patient's level of deep inspiration from outside of the treatment room. When a predetermined threshold is achieved, an interface box sends a logic signal to an interface board in either the CT simulator or the Varian 2100C/D LINAC. At the same time, when the threshold is reached, a signal from the relay output of the Micro‐P display goes to a pair of feedback glasses worn by the patient. The feedback glasses are ultraviolet‐blocking sunglasses with two frosted red LEDs mounted inside. The LEDs are connected to the relay output of the Micro‐P display such that when the trip point is reached and the beam is on, the LED lights turn on. In our technique, we use feedback glasses to help patients properly monitor breath‐hold and to improve the reproducibility of this maneuver.

### B. Clinical procedure

**Figure 3:**
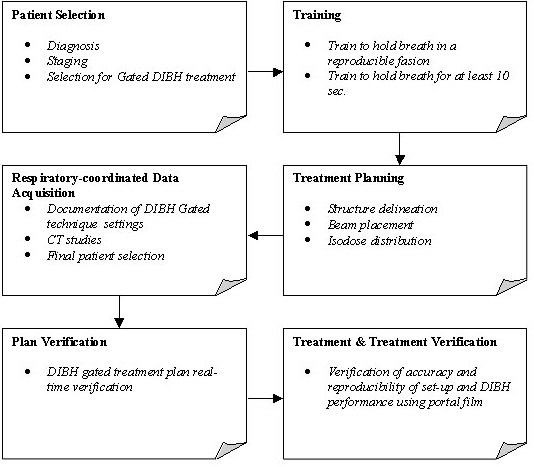
Block diagram of the deep inspiration breath hold (DIBH) gated clinical process

The block diagram in Fig. 3 summarizes our LPT‐based DIBH clinical process.

#### B.1 Patient training

Patients selected for DIBH treatment should have adequate pulmonary function, the ability to follow the procedure, and the ability to perform DIBH. Patients with significant cough, pain, or anxiety or with abdominal or shallow breathing patterns are not suitable for this technique. The DIBH maneuver should be performed at full lung capacity, and it demands certain skills from patients.

Patients are scheduled for the training session prior to the simulation. During the training session, they are coached by a radiotherapist on the reproduction of the same deep inspiration level during the simulation and subsequent treatment.

**Figure 4:**
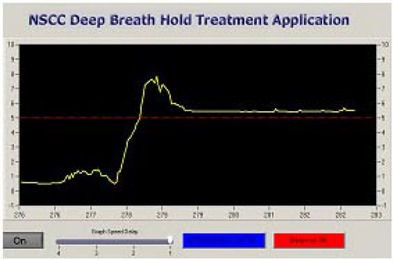
Deep inspiration breath‐hold signal

The same patient setup is used for all steps of the clinical process. Patients are placed in the supine position with both arms up above the head, with hands holding opposite wrists. A cushioned support is placed under the knees (Fig. 2). A MED‐TEC Vac‐Lok immobilization system is used for accurate and reproducible positioning of the patient throughout the simulation and treatment. A strap from the motion detector LPT is adjusted on the upper abdominal region of the patient. It is thin and narrow with minimum markings of 1 mm, which enables the therapist to position the patient reproducibly. The LPT connected to the Micro‐P device monitors the respiration level. The radiotherapist can estimate the level of the patient's respiration and the reproducibility of the DIBH maneuver from the graph on the display of the computer (Fig. 4). The graph represents the respiratory signal versus the elapsed time.

To set up the apparatus, the patient first takes and holds a deep breath. The tension on the strap is adjusted until the Micro‐P reaches the trip value, and the value on the strap is recorded. Adequate performance of the system and strap tension adjustment are checked by asking the patient to take a few regular breaths and by observing the breathing motion signal on the computer monitor. If the tension on the strap is not adjusted properly, the system will not track the respiration motion correctly, and it is necessary to repeat the adjusting procedure.

Patients are asked to practice the DIBH maneuver at home to increase the time of a breath‐hold and to improve the reproducibility level. We found that it is best to practice twice per day for at least 10 deep breath‐holds. Because stomach content can influence reproducibility of the organ positions, the patients are requested not to eat during the 2 h before simulation or treatment sessions.

#### B.2 CT simulation

In our technique, we use a CT study with multiple acquisitions. The studies were performed on a Picker PQ2000S AcQSim CT‐simulator. The CT imaging system is capable of operating in conjunction with a contrast injector, which allows a patient undergoing the DIBH maneuver to send a signal that triggers the CT scanner acquisitions.

To perform a single helical scan triggered by a signal present at the injector interface, the system operators select the injector trigger option via the Operator Communications interface at the CT console and then begin the scan.

When the new study is requested from the Operator Communications interface (Opcom) software, the scan parameters, such as the requested kilovolts, milliamperes, slice thickness, filter, and compensator, are sent to the X‐ray controller (the XSC+) in preparation for the scan. When all conditions are satisfied, the console's Start button will light green. Depressing the Start button will not actually start the scan but will set the injector latch (INJSTRT, injector start) within the scanner. The CT scanner will then wait for the trigger from the injector line (INJIACT, injector activate).

The LPT, adjusted to an upper abdominal region, tracks the changing body circumference and produces an analog signal, which is fed into the Micro‐P device. The Micro‐P interface box converts the analog signal to a digital signal, and when the signal from the LPT reaches a predetermined level, an interface box sends a logic signal to the injector port J700 of the Picker PQ2000S AcQSim CT simulator, and acquisition is started. At the end of the study a signal (INJSTOP, injector stop) is issued, and acquisition is terminated.

Spiral CT scan acquisitions are performed with a slice thickness of 0.3 cm in the tumor region and 0.5 cm in the rest of the scanning region. The number of acquisitions to encompass the entire superior‐inferior extent is determined by the time interval during which patients can maintain a breath‐hold. During training sessions, the average time of breath‐hold is estimated and is used for planning the time of acquisitions in CT studies. For an average breath‐hold time of 10 s, approximately eight acquisitions per study are done (~10 slices per acquisition). After the first study is accomplished, the patient takes a few regular breaths and then repeats the DIBH maneuver. Once the signal from the LPT reaches the threshold level, the second scanning study starts. The procedure is repeated until the entire planned thorax region is scanned.

**Figure 5:**
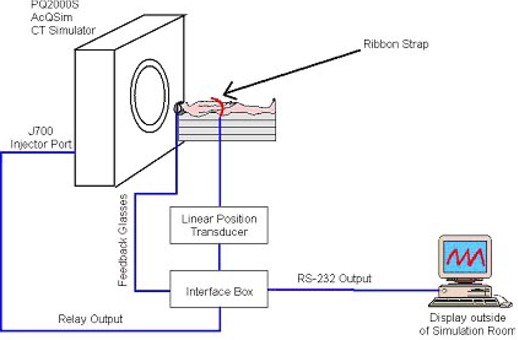
Schematic diagram of the CT simulation setup for the deep inspiration breath‐hold

The schematic diagram of the CT simulation setup of the DIBH using an LPT is presented in Fig. 5.

#### B.3 Plan verification

Subsequent to CT scanning, the target volume/structure delineation and 3D treatment planning are performed. For our DIBH‐gated technique plan verification, we use a Phillips SLS 6507 simulator. For the verification procedure, the patient is positioned in exactly the same manner as for the treatment setup and CT simulation. During plan verification, the patient is imaged in the anterior‐posterior and lateral directions while the patient's breathing cycle is monitored by the LPT device. Fluoroscopic studies during the patient's normal breathing can be performed to quantify the relationship between the respiratory volume and diaphragm movement. This allows the estimation of the position of the diaphragm and other thoracic organs during the respiration cycle. Fluoroscopy studies have been found to be extremely useful for studying the direction and magnitude of organ motion.^(6)^ ^,^ ^(8)^ ^,^ ^(10)^

In the case of the DIBH technique, intrafraction tumor movement during breath‐hold should be considered. Despite the fact that the breath‐hold technique nearly immobilizes the tumor, there is still residual motion of up to 10% of the normal breathing cycle movement of the tumor.^(15)^

### B.4 Treatment and treatment verification

**Figure 6:**
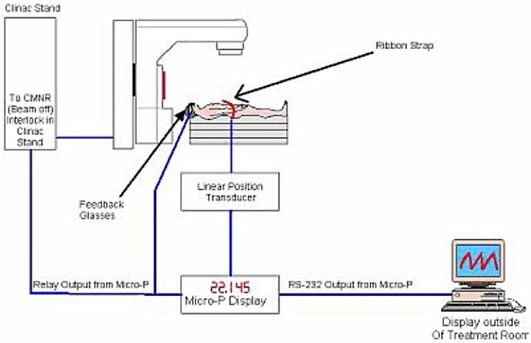
Setup for DIBH treatment at the LINAC

In our clinic, the DIBH gated radiation treatment is performed on a Varian Clinac 2100 C/D. The setup of the LINAC is shown in Fig. 6. In order to reduce the required breath‐hold time for patients, the dose rate is chosen as 600 monitor units per minute. When a predetermined level of a deep inspiration is reached and the LPT voltage crosses the threshold value, a Micro‐P interface box sends a signal to the Clinac console (MHOLDOFF). When the relay output is activated in the Micro‐P during the DIBH maneuver, the MHOLDOFF signal is then driven high and the beam is turned on. When the patient terminates the DIBH maneuver, the relay is deactivated and the MHOLDOFF signal stays low.

Patients are trained to perform breath‐holds for at least 10 s. This means that the entire treatment can be delivered within 1 to 3 breath‐holds. Once the beam is gated, the patient is instructed to breathe regularly. The beam can be held off for a maximum of 15 s. If the beam is held off for more than 15 s, a DPSN (dose position) interlock will occur. It was shown with staff volunteers and patients that 15 s is sufficient time for taking a few regular breaths and for preparing for the next DIBH maneuver. When the patient is ready for the next breath‐hold, he or she takes and holds a deep breath, and the whole process is repeated.

Before each treatment a portal image is taken under breath‐hold for each field to verify the reproducibility of the DIBH maneuver. Portal images are taken daily during the first week of treatment and weekly thereafter.

## III. RESULTS AND DISCUSSION

Our system is based on the use of a new external motion sensor, an LPT, to monitor patient breathing‐induced organ motion by tracking changes in the patient's abdominal cross‐sectional area. The LPT is a patient‐friendly, easy‐to‐operate, and cost‐effective device. However, the implementation of our technique has some limitations. First, it is not suitable for all patients with thoracic‐abdominal lesions. Patients with shallow and abdominal breathing patterns have to be excluded because the LPT sensor cannot track adequately in a reproducible manner if the chest displacements during normal breathing and breath‐hold are distinctly different.

As found by previous users of breathing‐synchronized radiotherapy,^(8)^ our first DIBH patient experience shows a significant increase in treatment time compared to the conventional radiotherapy (15 min to 30 min). It is expected that this time will decrease as the therapists get used to the technique, in particular, with more experience in adjusting the sensor during patient setup.

The DIBH maneuver used in our technique allows sparing of normal tissue due to lung expansion, thereby moving healthy lung tissue away from the primary beam and achieving tumor immobilization. One of the most significant benefits of using DIBH compared to gated, free‐breathing approaches is avoiding any discrepancy between the internal organ movement and external chest movement. Implementation of DIBH requires at least one 30‐min patient training session prior to simulation. During this session, the patient is trained to perform the DIBH maneuver reproducibly and to increase the time of a breath‐hold.

Various authors have shown that the feedback of the breathing signal to the patient plays a positive role in signal reproducibility.^(16)^ In our approach, we use a set of feedback glasses with built‐in LEDs to allow patients to cooperate fully in the process and to provide assurance that the system is properly monitoring their breathing. From our first patient experience, it was demonstrated that this is a very effective method for enabling the patient to maintain a steady breath‐hold and to improve the duration of the breath‐hold.

At the time of writing, the first patient has been treated using the gated DIBH radiotherapy technique. The patient had a 3‐cm mass in the superior segment of the right lower lobe as well as ipsilateral mediastinal lymphadenopathy. The patient was involved in gated treatment during phase II. In phase I, he received conformal radiotherapy treatment. The patient was trained for the DIBH maneuver, was very cooperative, and performed breath‐holds very well. The CT simulation with a thoracic scan, which included all lung tissue, was performed during breath‐hold maneuvers.

**Figure 7:**
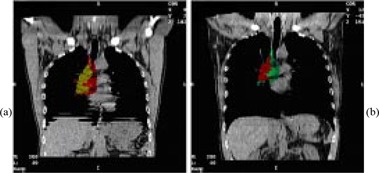
(a) Coronal slice reconstruction for free breathing during image acquisition (phase I). Gross tumor volume (GTV), yellow; PTV, red. (b) Coronal slice reconstruction for DIBH maneuver during image acquisition (phase II). GTV, red; PTV, green. (CT studies for two phases were performed at different times. That is why the colors chosen for outlining the target are not similar.)

Comparison of coronal slice reconstructions from phase I with free breathing during image acquisition (Fig. 7(a) and phase II with the DIBH maneuver performed during image acquisition (Fig. 7(b) shows significant blurring effects on the free‐breathing image related to periodical respiratory motion of anatomical structures.

The radiation oncologist has decided not to reduce the PTV margins and did not change the field size for the first patient. However, even with the same parameters, the treatment planning has shown a 3% reduction in V20. Using the DIBH technique, the total lung volume increased by 26%, from 5000 cm^3^ to 6300 cm^3^.

**Figure 8:**
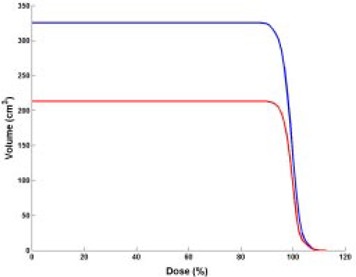
Cumulative dose‐volume histograms (DVHs) for phase II performed with the DIBH maneuver. Red: DVH for phase II PTV with the same CTV to PTV margin as phase I; blue: DVH for phase II PTV if margin had been reduced by 0.5 cm. (The patient was treated with the DIBH gated technique during phase II only; for phase I he had conformal radiotherapy treatment.)

If tighter PTV margins had been used to exploit the gated DIBH radiotherapy technique's full capability to immobilize the tumor and improve setup accuracy, a significant amount of healthy lung tissue would have been excluded from the high‐dose region. The dose‐volume histograms in Fig. 8 illustrate the possible normal tissue volume reduction of 112 cm^3^ if clinical target volume (CTV) to PTV margins had been reduced by 0.5 cm. Due to the sparing of this volume of normal tissue, a dose escalation to the target volume would have been possible with the same risk of normal tissue complications.

## IV. CONCLUSION

We have developed and implemented a low‐cost, automated deep inspiration breath‐hold gated technique using a new external motion sensor (a linear position transducer) for treatment of patients with thoracic lesions at the Nova Scotia Cancer Centre in Halifax. LPT has been used for tracking the organ position in the thoracic region during a breath‐hold. It was demonstrated that the sensor has a rapid response and produces accurate and reproducible signals. Using accelerator gating, the treatment can be delivered automatically whenever the patient achieves a preset deep‐inspiration breath level. Our work confirms the feasibility of the gated DIBH radiotherapy technique in treatment of thoracic lesions and its ability to dramatically improve the accuracy of treatment for moving tumors.

The technique is useful for patients with good lung function, no significant cough, and low anxiety. Because of these criteria, a large number of patients are not suitable for the DIBH gated treatment; however, those who are suitable can derive considerable benefits from this treatment technique. Despite some of the limitations, our technique allows us to minimize the motion of the tumor, reduce the lung volume in the high‐dose region, and gate the beam automatically. Our first patient experience result is in agreement with the results obtained by other authors of the DIBH technique. On the next phase of our work we will gather more clinical data of changes in the target and irradiated volumes of patients treated with the DIBH radiotherapy technique to estimate the technique's effectiveness and potential to reduce toxicity and improve tumor control. Although the present work uses an LPT as a sensor, the technique can be readily adapted to other sensors as well.

## ACKNOWLEDGMENTS

The authors thank Dr. Liam Mulroy, Dr. Wladislawa Swajna, Ian Porter, David Burgess, Lisa Bourne, Helen Burgess, Joleen Orr, CarolAnn Jessiman, Heather Edstrom, Dr. James Robar, and Jason Schella for their help during the development and implementation of this project.
